# Feature selection for global tropospheric ozone prediction based on the BO-XGBoost-RFE algorithm

**DOI:** 10.1038/s41598-022-13498-2

**Published:** 2022-06-02

**Authors:** Biao Zhang, Ying Zhang, Xuchu Jiang

**Affiliations:** 1 School of Computer Science, Liaocheng University, Liaocheng, 252000 China; 2 School of Statistics and Mathematics, Zhongnan University of Economics and Law, Wuhan, 430073 China

**Keywords:** Climate sciences, Ecology, Environmental sciences, Environmental social sciences, Computational science, Computer science

## Abstract

Ozone is one of the most important air pollutants, with significant impacts on human health, regional air quality and ecosystems. In this study, we use geographic information and environmental information of the monitoring site of 5577 regions in the world from 2010 to 2014 as feature input to predict the long-term average ozone concentration of the site. A Bayesian optimization-based XGBoost-RFE feature selection model BO-XGBoost-RFE is proposed, and a variety of machine learning algorithms are used to predict ozone concentration based on the optimal feature subset. Since the selection of the underlying model hyperparameters is involved in the recursive feature selection process, different hyperparameter combinations will lead to differences in the feature subsets selected by the model, so that the feature subsets obtained by the model may not be optimal solutions. We combine the Bayesian optimization algorithm to adjust the parameters of recursive feature elimination based on XGBoost to obtain the optimal parameter combination and the optimal feature subset under the parameter combination. Experiments on long-term ozone concentration prediction on a global scale show that the prediction accuracy of the model after Bayesian optimized XGBoost-RFE feature selection is higher than that based on all features and on feature selection with Pearson correlation. Among the four prediction models, random forest obtained the highest prediction accuracy. The XGBoost prediction model achieved the greatest improvement in accuracy.

## Introduction

Ozone (o_3_) is a toxic greenhouse gas. Stratospheric ozone can protect life on the Earth's surface from ultraviolet radiation, but tropospheric ozone, as the second largest pollutant in the atmosphere, is harmful to human health and vegetation^1,2^. The two main factors that produce high-quality ozone pollution are meteorological conditions and the concentration of ozone precursors^3^. Meteorological conditions are one of the most important factors affecting the concentration of ozone near the ground. The important influencing factors in various meteorological conditions include solar ultraviolet radiation, relative humidity, wind direction and wind speed, which affect photochemical reaction conditions^4^. Ozone is mainly a secondary pollutant produced by photochemical reactions of NOx and VOCs, so it is closely related to the concentration of precursor substances. The emission sources of precursor substances NOx and VOCs can be divided into anthropogenic emission sources and natural emission sources. Anthropogenic emission sources are mainly formed by the production process of petrochemical-related industries, product consumption behavior and vehicle exhaust emissions^5^. To better prevent and address the threat of tropospheric ozone pollution, it is very important to establish an accurate and reliable prediction model and understand the key factors affecting ozone concentration^6–9^. Data-driven atmospheric chemistry research has begun to be combined with machine learning, which is widely used in the prediction of pollutant concentrations^10^. Ortiz-García et al.^11^ carried out the prediction of ozone concentration based on the concentration data of this site, the concentration data of neighboring sites, and meteorological variables through support vector machine regression, and in the process of using different factors for prediction, the prediction results were significantly different from those at other times. For different moments, the best moment data of different factors are used to form a dataset for prediction. Dong et al.^12^ combined the regionality and periodicity of ozone and proposed an ozone prediction model PCA-PSO-SVM that integrates temporal and spatial characteristics, using Hangzhou meteorological data and pollutant data to predict the daily maximum 8-h average concentration. The model showed better prediction accuracy and applicability. Liu et al.^13^ combined the observation data of MDA8 (maximum daily 8-h average ozone), combined with parallel ozone inversion, aerosol reanalysis, meteorological parameters and land use data, and established a national MDA8 prediction model based on XGBoost. The external test of the regional measured data from 2005 to 2012 and the national data in 2018 shows that the model has strong robustness and reliability in the prediction of historical data.

By combining the ozone formation mechanism^14^, the dataset in this study establishes features including site locations and environmental information and aggregates and averages multiyear ozone concentration data as an indicator of long-term ozone concentration. The longer aggregation period averages out short-term weather fluctuations, making it immune to short-term weather and unusual emissions. In the short-term prediction of ozone, historical emission data, meteorological monitoring data and air pollutant monitoring data are mostly used to construct features to predict the concentration. However, historical meteorological monitoring data and atmospheric pollutant data are not suitable for the prediction of ozone concentrations on a longer time scale. Because ozone concentrations are determined by many interrelated effects, such as precursor emissions, land use, land cover, and climatic conditions^15^, many of these factors and their interconnections cannot be accurately quantified, so we construct features by using environmental information as proxy variables that are associated with the mechanisms of ozone formation to predict long-term ozone concentrations. For example, one of the variables in the dataset is the climatic zone in which the site is located. Ozone is affected by weather, and to better represent the effect of weather on ozone concentrations on a long time scale, we can use the climate zone as an indicator of weather on longer time scales. In addition, the main sources of ozone precursor emissions include human activities such as transportation and emissions from industrial activities. Population density and average nighttime light intensity observed from space are used as proxies for human activity and industrial production. This method makes it possible to predict the long-term ozone concentration of the site by obtaining environmental information about the site itself.

In previous studies, many complex and interrelated features, including precursor emissions, land cover, geographic location, and climate change, were all input into the model for prediction, and there was a lack of feature extraction in the ozone concentration prediction model. Since not all factors are related to predictor variables, proper selection of variables can improve the prediction accuracy; inputting too many features, especially those with strong correlation into the model, will reduce the efficiency and accuracy of training, increasing the model training burden^16^. Under the condition of ensuring prediction accuracy, on the one hand, the model dimension can be reduced, and the efficiency of the algorithm can be improved. On the other hand, extracting the main variables in the model by feature selection is conducive to further understanding the key factors affecting ozone concentration and provides reference value for the prevention and response to ozone threats^17^. In the study of feature selection for air quality indicators and pollutant forecasting, Domańska et al.^18^ used backward elimination to extract important variables that affect ozone concentration by determining the validity of each input feature. The results show that the subset of parameters found by the reverse elimination feature selection method provides the greatest prediction accuracy. Hui et al.^19^ used spatial correlation analysis, correlation analysis (mutuality) and binary gray wolf optimization (BGWO) in the spatial prediction problem for AQI (air quality indicator) 3 stage-by-stage extraction of spatiotemporal features. Sethi et al.^20^ used the causality-based linear method to select the main factors affecting PM2.5 content. Compared with the prediction model without feature selection, it is found that the model after feature selection obtains higher prediction accuracy.

## XGBoost-RFE algorithm

### XGBoost model

XGBoost was first proposed by Chen et al.^21^ in 2014. It is a decision tree based on gradient lifting (GBDT) and is an efficient system implementation of the boosting method. In the XGBoost model, the objective function is expanded by second-order Taylor, and the optimization problem of the objective function is transformed into solving the minimum value of the quadratic function. At the same time, tree complexity is added to the objective function as a regularization term to improve the generalization performance of the model. The objective function of the model is:

1 $$\begin{array}{*{20}c} {O = \mathop \sum \limits_{i = 1}^{n} L\left( {y_{i} ,\hat{y}_{i} } \right) + \mathop \sum \limits_{k = 1}^{K} \Omega \left( {f_{k} } \right)} \\ \end{array}$$

where *n* is the sample size; *y*_*i*_ is the true value of the *i*-th sample; $\hat{y}_{i}$ is the predicted value of the *i*-th sample; $L\left( {y_{i} ,\hat{y}_{i} } \right)$ is the difference between the real value *y*_*i*_ and the predicted value $\hat{y}_{i}$; $\Omega \left( {f_{k} } \right)$ is the tree complexity; and *K* is the number of features.

Taylor second-order expansion of the objective function:

2 $$\begin{array}{*{20}c} {O^{t} = \mathop \sum \limits_{i = 1}^{n} \left[ {g_{i} f_{t} \left( {x_{i} } \right) + \frac{1}{2}h_{i} f_{t}^{2} \left( {x_{i} } \right)} \right] + \Omega \left( {f_{k} } \right) + C} \\ \end{array}$$

The first and second derivatives of the loss function are defined as *g*_*i*_, *h*_*i*_; $f_{t} \left( {x_{i} } \right)$ is the structure value of tree *x*_*i*_ in the *t*-th iteration.

The definition of a tree is:

3 $$\begin{array}{*{20}c} {f_{t} \left( x \right) = w_{q\left( x \right)} ,\;\;w \in R^{T} ,\;\;q:R^{d} \to \left\{ {1,2, \ldots ,T} \right\}} \\ \end{array}$$

where *q* represents the structure of the tree: Map input samples $x_{i} \in R^{d}$ to leaf nodes; *T* is the number of leaf nodes; and *w* is a one-dimensional vector with length *T*, which represents the weight of leaf nodes.

The complexity of the tree is obtained by weighting the number of leaf nodes of the tree and the *L*2 norm of the node weight vector, which is defined as:

4 $$\begin{array}{*{20}c} {\Omega \left( {f_{t} } \right) = \gamma T + \frac{1}{2}\lambda \mathop \sum \limits_{j = 1}^{T} w_{j}^{2} } \\ \end{array}$$

The objective function can be rewritten as:

5 $$\begin{array}{*{20}c} {O^{t} = \mathop \sum \limits_{j = 1}^{t} \left[ {G_{j} w_{i} + \frac{1}{2}\left( {H_{j} + \lambda } \right)w_{j}^{2} } \right] + \gamma T} \\ \end{array}$$

$$\begin{aligned} & = - \frac{1}{2}\mathop \sum \limits_{j = 1}^{T} \frac{{G_{j}^{2} }}{{H_{j} + \lambda }} + \gamma T \\ G_{j} & = \mathop \sum \limits_{{i \in I_{j} }} g_{i} ,\quad H_{j} = \mathop \sum \limits_{{i \in I_{j} }} h_{i} \\ \end{aligned}$$

where *I*_*j*_ is the sample set of the *j*-th leaf node, $\lambda$ and $\gamma$ is the weight factor.

### Importance metrics

The calculation methods of feature importance in the XGBoost model are mainly three ways: weight (the number of times a feature is used to split the data across all trees), gain (the average gain across all splits the feature) and cover (the average coverage across all splits the feature). In this study, the weight is used as a measure of feature importance.

### Recursive feature elimination algorithm based on XGBoost

**Figure 1:**
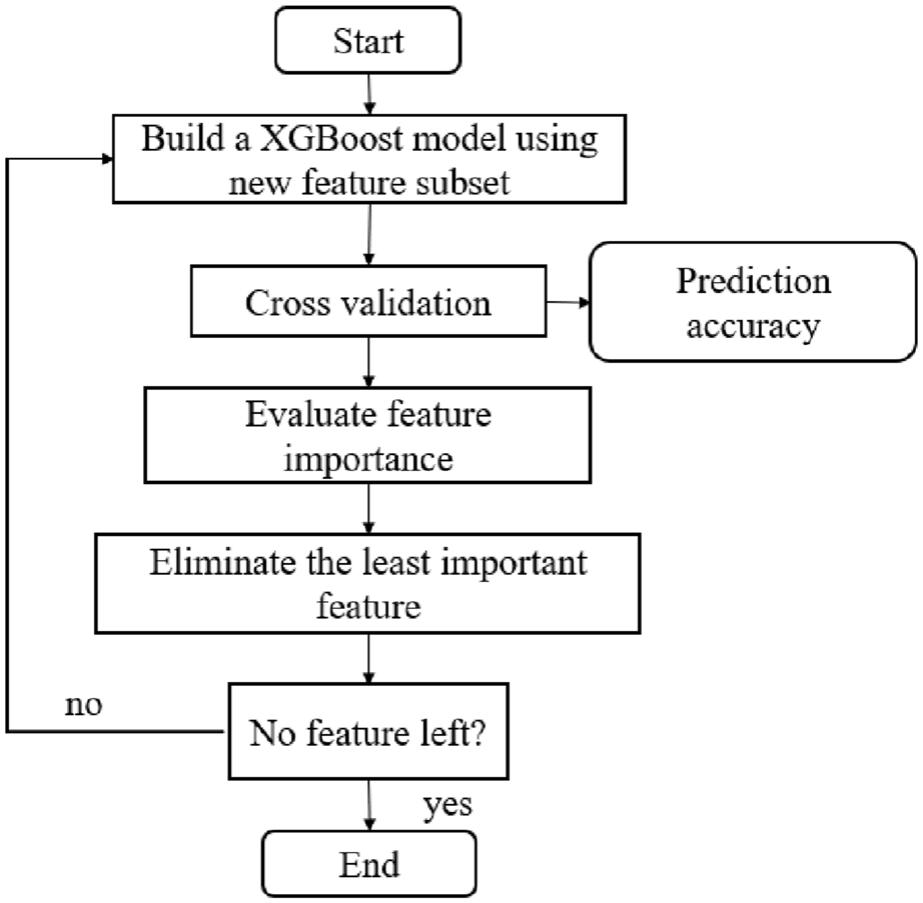
Workflow of XGBoost-RFE.

Recursive feature elimination (RFE) is a sequential backward selection algorithm belonging to Wrapper^22^, which uses a specific underlying algorithm to select features by recursively reducing the size of the feature set. RFE was first proposed by Guyon I^23^ based on the SVM model, which achieved very good results in the process of gene selection and subsequently became a widely used method in gene selection research. XGBoost-RFE takes XGBoost as an external learning algorithm for feature selection, sorts the importance of features in each round of feature subset, and eliminates the features corresponding to the lowest feature importance to recursively reduce the scale of the feature set, and the feature importance is constantly updated in each round of model training. Based on the selected feature set, this study uses cross validation to determine the feature set with the lowest average score based on the mean absolute error (MAE). The algorithm flow chart is shown in Fig. 1.

XGBoost-RFE algorithm flow:

1. XGBoost is trained from the training set *T* containing all samples, and the fivefold cross validation method is used to evaluate the prediction accuracy of XGBoost based on the new feature subset after each round of feature elimination based on the mean absolute error (MAE);
2. Calculate and sort the importance ${\text{IM}}\left( \propto \right)$ of each feature $\propto$ in the feature set based on gain (Gain);
3. According to the backward selection of the sequence, delete the feature with the lowest feature importance, and repeat the remaining feature subset for 1–2 until the feature subset is empty. According to the cross-validation results of each feature subset, the feature subset with the highest prediction accuracy is determined.

## Bayesian optimized XGBoost-RFE

### Bayesian optimization

This study performs feature selection based on XGBoost-RFE. Since the feature selection process involves the selection of the underlying model hyperparameters, different hyperparameter combinations will lead to differences in the feature subset selected by the model, so the obtained feature subset may not be the optimal solution. To obtain the optimal subset, this study combines the Bayesian optimization algorithm to adjust the parameters of the model and then obtains the optimal feature subset. Currently, the common parameter optimization methods are the grid search method and random search method. The grid search method adopts the method of traversing the parameter set, which is inefficient. Faced with a model with a large parameter space and a large number of parameters, it is easy to cause dimension explosion, which is not feasible. The random search method is used to randomly optimize the parameters, and it is easy to miss the optimal solution. This problem can be effectively addressed by Bayesian optimization (BO)^24^. BO has high optimization efficiency and can find an excellent parameter set with less computational cost^25–27^.

The Bayesian optimization algorithm establishes a probabilistic surrogate model of the objective function based on the historical evaluation results of the objective function and makes full use of the previous evaluation information when selecting the next set of hyperparameters, reducing the number of hyperparameter searches, and the obtained hyperparameters are also most likely to be optimal, thereby increasing the accuracy of the model. The Bayesian optimization algorithm is based on Bayes' theorem, obtains the next-most-potential evaluation point x by maximizing the acquisition function, evaluates the objective function value y, and adds the newly obtained (x, y) to the known evaluation. In the set of points, update the probability proxy model, and iterate to obtain the optimal solution^28^. Bayesian optimization mainly includes two parts: a probabilistic surrogate model and an acquisition function (AC). A probabilistic surrogate model is a probabilistic model used to represent an unknown objective function. Among them, the Gaussian process has a strong fitting function performance (Gaussian process, GP) and is the most widely used.

The Gaussian process is the parameter combination for the XGBoost model that needs to be optimized, namely:

6 $$\begin{array}{*{20}c} {\left\{ {\begin{array}{*{20}l} {f\left( x \right)\sim gp\left( {m\left( x \right),k\left( {x,x^{\prime } } \right)} \right)} \hfill \\ {m\left( x \right) = E\left[ {f\left( x \right)} \right]} \hfill \\ {k\left( {x,x^{\prime } } \right) = E\left[ {\left( {f\left( x \right) - m\left( x \right)} \right)\left( {f\left( {x^{\prime } } \right) - m\left( {x^{\prime } } \right)} \right)} \right]} \hfill \\ \end{array} } \right.} \\ \end{array}$$

where *m*(*x*) is the mean function, $k\left( {x,x^{\prime}} \right)$ is the covariance function, and the prior distribution of the unknown function can be expressed as $p\left( {f_{1:t} {|}D_{1:t} } \right)\sim N\left( {0,K_{t} } \right)$, where $f_{1:t}$ is the set of *f* values corresponding to the sampling points, and $K_{t}$ is the covariance matrix formed by the covariance function.

The acquisition functions generally include PI, EI, and UCB. This study chooses the UCB function based on the confidence interval strategy as the acquisition function. Choosing a strategy based on confidence intervals (UCB) is the next evaluation point for:

7 $$\begin{array}{*{20}c} {x_{t + 1} = argmax\mu_{t} \left( x \right) + \sqrt {\beta_{t} } \sigma_{t} \left( x \right)} \\ \end{array}$$

where $v^{*}$ is the optimal value of the current objective function, $\phi \left( \cdot \right)$ is the standard normal distribution cumulative density function, and $\xi$ is the balance parameter. By adjusting $\xi$, we can avoid falling into the local optimum and realize the global search for the optimum value.

### Bayesian optimized XGBoost-RFE feature selection

XGBoost-RFE uses XGBoost as the underlying learner for recursive feature elimination and adopts the sequential backward selection method to sort and select features according to the feature importance measure output by XGBoost. However, the XGBoost model has many hyperparameters, and using different hyperparameter combinations can lead to differences in the optimal subsets obtained by the model during feature selection. Using inappropriate hyperparameters may result in a subset that is not optimal. Therefore, Bayesian optimization is used to optimize the recursive elimination model with XGBoost as the underlying learner to search for hyperparameters and corresponding optimal subsets that can minimize the cross-validation error after dimensionality reduction.

**Figure 2:**
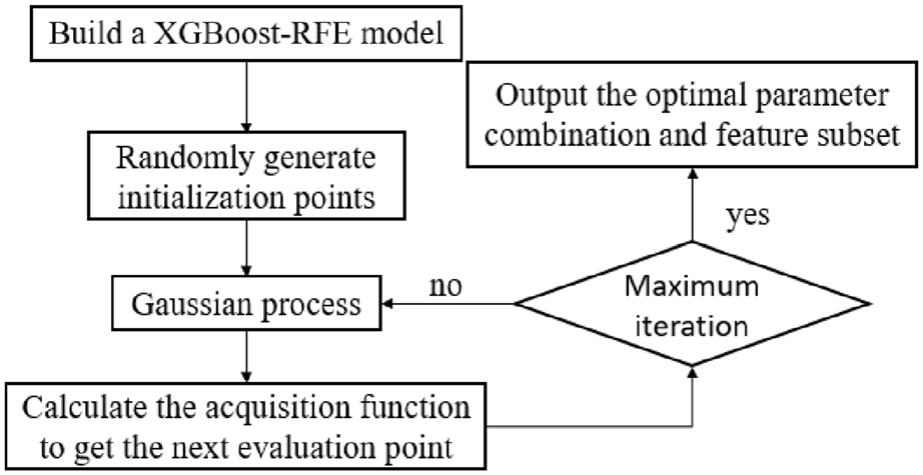
BO-XGBoost-RFE.

The accuracy of the optimal feature subset obtained by XGBoost-RFE cross-validation is used as the objective function, and different parameter combinations of the model are used as independent variables to form a surrogate model framework for Bayesian optimization iterations. The parameter optimization process of the XGBoost-RFE model is shown in Fig. 2.

The BO-XGBoost-RFE process is:

1. Initialize the XGBoost model parameters and the range of hyperparameters to generate random initialization points. The training set and initialization parameters are used as the input variables of the Gaussian model in Bayesian optimization, the cross-validation results of XGBoost-RFE under each set of parameters are used as the objective function, and the parameters are modified to improve the Gaussian model;
2. Select the parameter combination points to be evaluated in the revised Gaussian model so that the acquisition function is optimal, the Gaussian model is closer to the true distribution of the objective function than the other parameter combination points, and the optimal parameter combination is obtained:
3. Input the parameter combination into the model for training, output the corresponding parameter combination and the prediction error of the model ($x,f\left( x \right))$, add the newly collected samples $\left( {x,f\left( x \right)} \right)$ to the historical sampling set, and update the Gaussian model;
4. When the maximum number of iterations is reached, the model update is stopped, and the optimal sampling point and the corresponding optimal subset are output.

## Prediction of ozone concentration based on BO-XGBoost-RFE

### Experimental data

**Figure 3:**
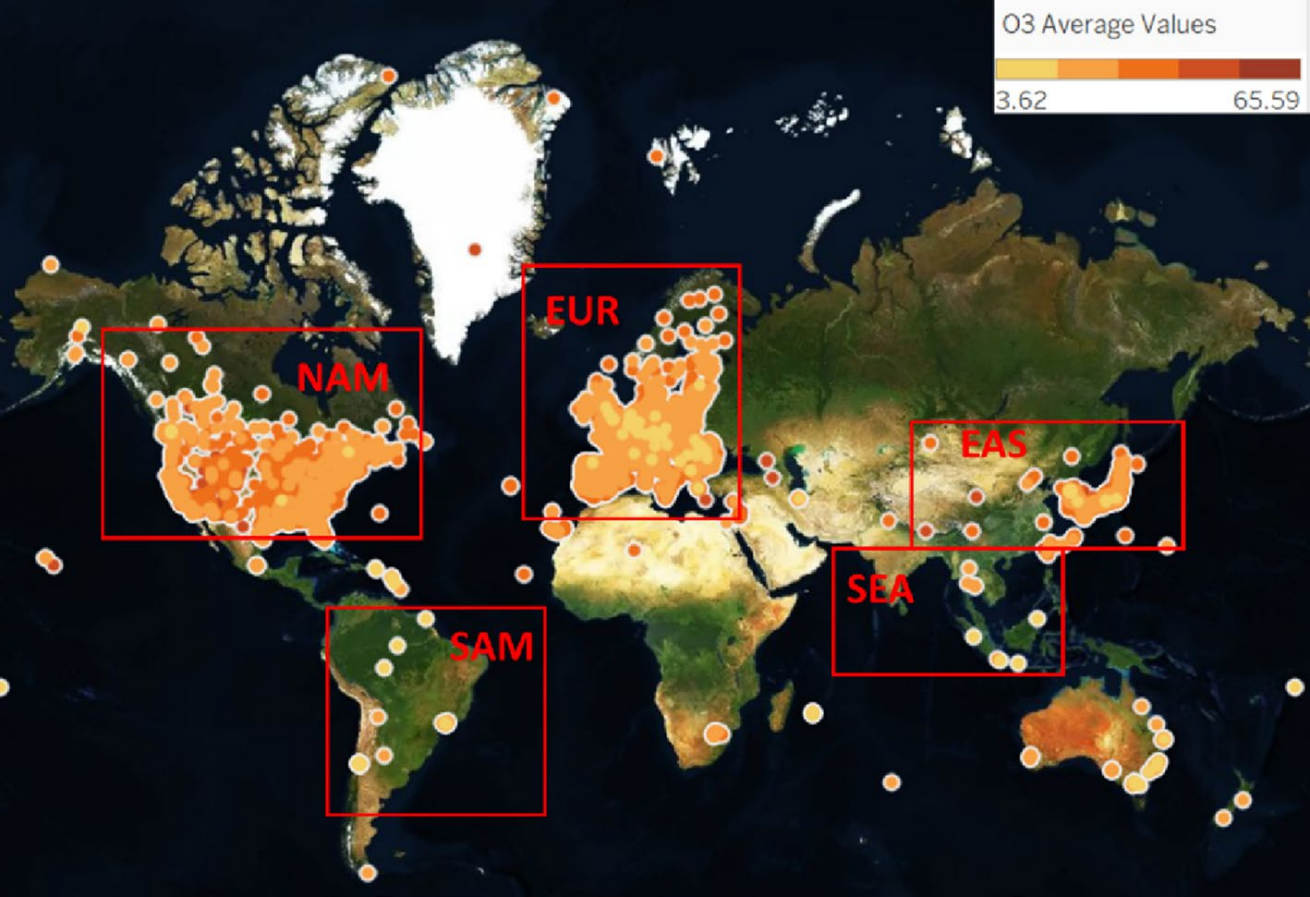
Global distribution of monitoring sites.

The dataset used in this study is the global long-term air quality indicator data of 5577 regions from 2010 to 2014 extracted by Betancourt et al.^14^ based on the TOAR database (https://gitlab.jsc.fz-juelich.de/esde/machine-learning/aq-bench/-/blob/master/resources/AQbench_dataset.csv)^29^. As shown in Fig. 3, the monitoring sites include 15 regions, including EUR (Europe), NAM (North America), and EAS (East Asia), and are mainly distributed in NAM (North America), EUR (Europe) and EAS (East Asia). The dataset mainly includes the geographical location information of the monitoring site, such as longitude and latitude, the area to which it belongs, altitude, etc., and the site environment information, such as population density, night light intensity, and vegetation coverage. Since it is difficult to directly quantify factors such as the degree of industrial activity and the degree of human activity, environmental information such as the average light intensity at night and population density are used as proxy variables for the above factors. The ozone indicator records the hourly ozone concentration from air quality observation points in various regions and aggregates the collected ozone time series in units of one year into one indicator. Using a longer aggregation period can be used to average short-term weather fluctuations. The experimental data have a total of 35 input variables, including 4 categorical attributes and 31 continuous attributes. The predictor variable is the average ozone concentration in each region from 2010 to 2014. The specific variable names and descriptions^14^ are shown in the supplementary materials. A total of 4/5 of the total samples were used as the training set, and 1/5 were used as the test set.

### Results of BO-XGBoost-RFE

**Figure 4:**
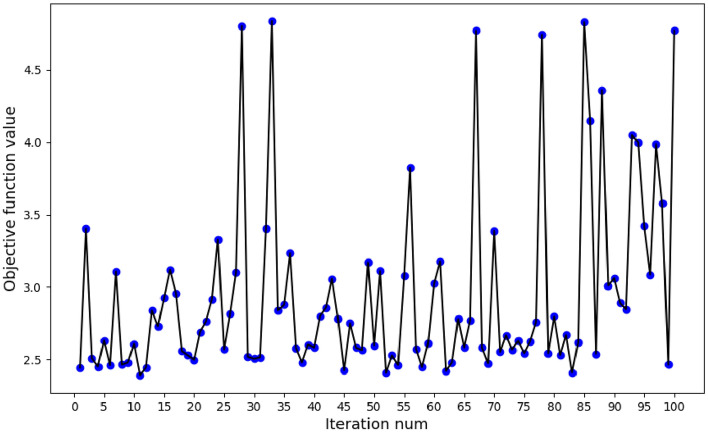
Iterative process of Bayesian optimization.

According to the XGBoost-RFE algorithm for feature selection, XGBoost-RFE combined with the cross-validation method is used to calculate the selected feature set in each RFE stage for fivefold cross-validation, and the mean absolute error (MAE) is used as the evaluation criterion to finally determine the number of features with the lowest mean absolute error (MAE). At the same time, the Bayesian optimization algorithm is used to adjust the hyper-parameters of XGBoost-RFE, and then the feature subset with the lowest cross-validation mean absolute error (MAE) is obtained. The main parameters of the XGBoost model in this article include the learning_rate, n_estimators, max_depth, gamma, reg_alpha, reg_lambda, colsample_bytree, and subsample. All parameters used in the model are shown in the supplementary material. Within the given parameter range, the Bayesian optimization algorithm is used, the mean absolute error (MAE) of the XGBoost-RFE fivefold cross-validation is used as the objective function, and the number of iterations is controlled to be 100. We obtained the hyperparameter combination corresponding to the lowest MAE and the corresponding optimal feature subset. The iterative process of Bayesian optimization is shown in Fig. 4.

**Figure 5:**
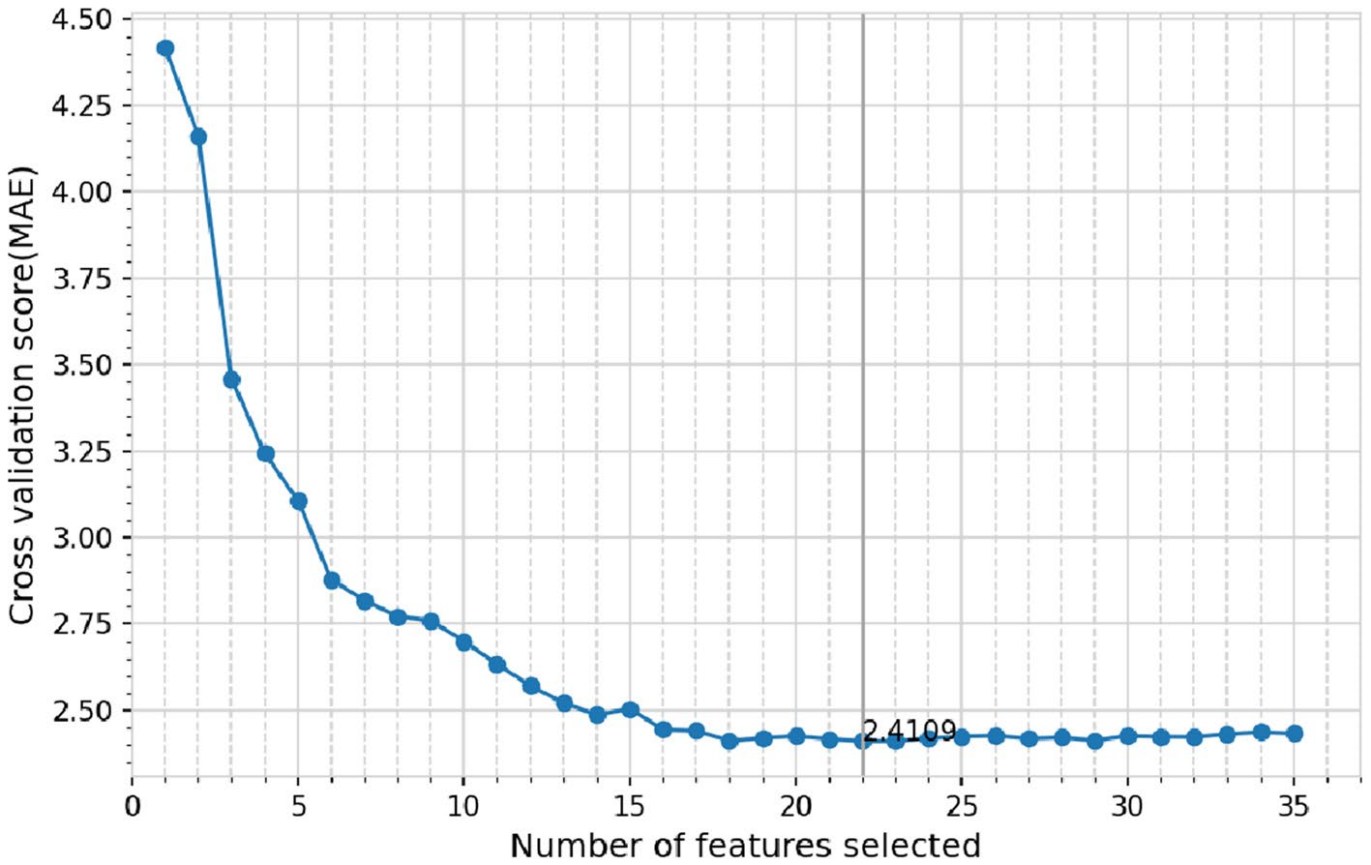
XGBoost-RFE feature selection results: Cross-validation MAE under optimal hyperparameter combination.

The parameter range and optimized value of XGBoost-RFE are shown in Table 1. The XGBoost-RFE feature selection results under the above optimized hyperparameters are shown in Fig. 5. The number of features in the feature subset with the lowest mean absolute error is 22, and the MAE is 2.410.

**Table 1:** Main hyper-parameter range and optimized value.

Hyper parameter	Range	Optimized value
Learning_rate	(0.001, 0.3)	0.0798
N_estimators	(50, 250)	134
Max_depth	(3, 15)	8
Min_child_weight	(1, 7)	4
Gamma	(0, 1)	0.676
Reg_alpha	(0, 1)	0.4873
Reg_lambda	(0, 1)	0.2451
Colsample_bytree	(0.1, 1)	0.7144
Subsample	(0.1, 1)	0.823

Additionally, the XGBoost-RFE feature selection model without Bayesian optimization is compared with the algorithm in this study. The default parameters of the underlying model XGBoost are set to learning_rate as 0.3, max_depth as 6, gamma as 0, colsample_bytree as 1, subsample as 1, reg_alpha as 1, and reg_lambda as 0. The comparison results are shown in Table 2. The results show that the XGBoost-RFE cross-validation MAE without parameter tuning is larger than that of the algorithm in this study, and the dimension of the feature subset obtained is also higher than that of the algorithm in this study.

**Table 2:** Comparison of MAE and feature num before and after BO.

Model	MAE	Feature number
BO-XGBoost-RFE	2.410	22
XGBoost-RFE	2.516	29

### Prediction results

To test the prediction accuracy of the prediction model with the optimal subset obtained by BO-XGBoost-RFE, three indexes, MAE, RMSE and *R*^2^, are used to evaluate the prediction results, and the expressions are as follows:

8 $$\begin{array}{*{20}c} {MAE = \frac{1}{n}\mathop \sum \limits_{i = 1}^{n} \left| {\left( {y_{i} - \widehat{{y_{i} }}} \right)} \right|} \\ \end{array}$$

9 $$\begin{array}{*{20}c} {RMSE = \sqrt {\frac{1}{n}\mathop \sum \limits_{i = 1}^{n} \left( {y_{i} - \widehat{{y_{i} }}} \right)^{2} } } \\ \end{array}$$

10 $$\begin{array}{*{20}c} {R^{2} = 1 - \frac{{\mathop \sum \nolimits_{i = 1}^{n} \left( {\widehat{{y_{i} }} - y_{i} } \right)^{2} }}{{\mathop \sum \nolimits_{i = 1}^{n} \left( {y_{i} - \overline{{y_{i} }} } \right)^{2} }}} \\ \end{array}$$

*n* indicates the number of samples, *y*_*i*_ is the true value, $\widehat{{y_{i} }}$ is the predicted value and $\overline{{y_{i} }}$ indicates the mean value of the predicted value.

The XGBoost-RFE feature selection algorithm based on Bayesian optimization in this study is compared with feature selection using full features and features selected by the Pearson correlation coefficient, which measures the correlation between two variables. In this study, the correlation with predictor variables was selected to be less than 0.1, and the variables with correlations greater than 0.9 were deleted to avoid multicollinearity.

XGBoost, random forest, support vector regression machine, and KNN algorithms were used to predict ozone concentration with full features, features selected by Pearson's correlation coefficient, and features based on BO-XGBoost-RFE. According to the evaluation indicators described above, the comparison of the prediction performance results of the three algorithms before and after dimensionality reduction can be obtained. The MAE, RMSE and R^2^ results of each prediction model are shown in Table 3.

**Table 3:** MAE, RMSE and R^2^ of each prediction model.

Model	After FS			After Pearson’s			All features		
	MAE	RMSE	R^2^	MAE	RMSE	R^2^	MAE	RMSE	R^2^
XGBoost	2.386	3.281	0.718	2.590	3.462	0.675	2.478	3.368	0.698
RF	2.374	3.206	0.720	2.500	3.380	0.690	2.407	3.266	0.710
SVR	2.676	3.631	0.659	2.912	3.871	0.583	2.677	3.620	0.636
KNN	2.801	3.808	0.606	2.873	3.837	0.601	2.846	3.834	0.601

Among the four prediction models, random forest has the lowest MAE and RMSE and the highest R^2^ based on three different dimensions of data and therefore has the best prediction performance. The prediction accuracy of all four prediction models based on Pearson correlation is lower than that based on BO-XGBoost-RFE, indicating that only selecting features by correlation cannot accurately extract important variables. Although the RMSE of the support vector regression model based on BO-XGBoost-RFE is slightly lower than the RMSE based on full features, the prediction accuracy of XGBoost, RF, KNN after feature selection of BO-XGBoost-RFE is higher than that based on full features and Pearson correlation. Among the four prediction models, random forest has obtained the highest prediction accuracy. The MAE based on BO-XGBoost-RFE is 5.0% and 1.4% lower than that based on the Pearson correlation coefficient and the full-feature-based model, and the RMSE is reduced by 5.1%, 1.8%, R^2^ improved by 4.3%, 1.4%. Additionally, the XGBoost model achieved the greatest improvement in accuracy. The MAE was reduced by 5.9% and 1.7%, the RMSE was reduced by 5.2% and 1.7%, and the R^2^ was improved by 4.9% and 1.4% compared with the Pearson correlation coefficient-based and full-feature-based models, respectively. This indicates that feature selection based on BO-XGBoost-RFE effectively extracts important features, improves prediction accuracy based on multiple prediction models, and has better dimensionality reduction performance.

**Figure 6:**
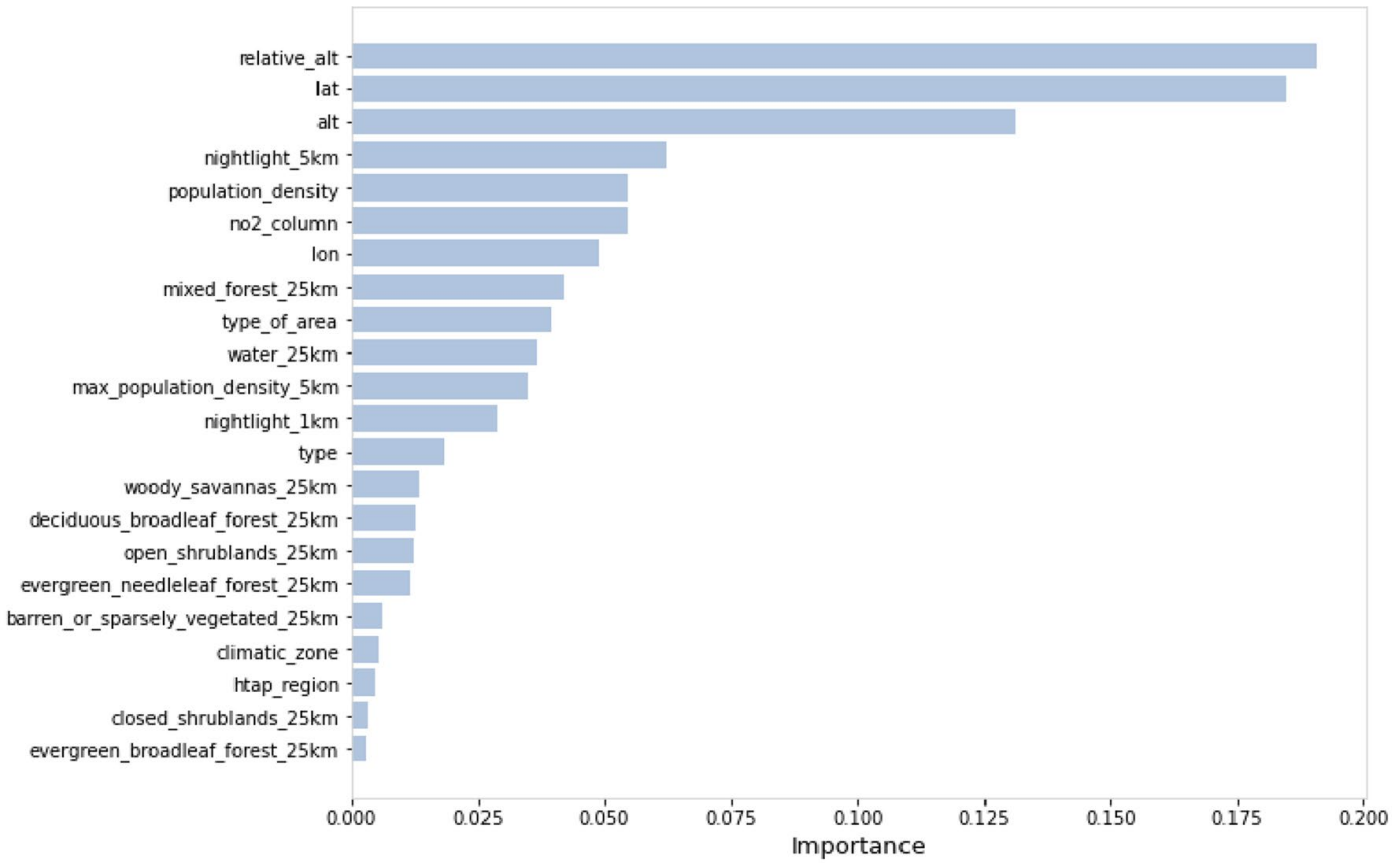
Feature importance in random forest.

Figure 6 shows the importance of each feature obtained by using the random forest prediction model, reflecting the degree of influence of each variable on the prediction results of the global multi-year average near-ground ozone concentration. The most important variables that affect the prediction results according to the ranking of feature importance are altitude, relative altitude, and latitude, followed by night light intensity within a radius of 5 km, population density and nitrogen dioxide concentration, while the proxy variables for vegetation cover have a relatively weak effect on the prediction of ozone concentration.

## Conclusion

This study uses the long-term air quality index observation data of 5,577 regions in the TOAR database from 2010 to 2014 and uses the geographic information and environmental information of monitoring sites as features to predict the long-term average ozone concentration. However, since the metadata contain a large amount of environmental information that affects the ozone concentration, the addition of irrelevant information will increase the noise of the data, thereby reducing the prediction accuracy, so it is necessary to select the features. In this study, a Bayesian optimization-based XGBoost recursive feature elimination method is proposed, which extracts important variables to improve the prediction accuracy of long-term ozone concentrations. The prediction accuracy based on the BO-XGBoost-RFE model is higher than that of the model based on all features and feature selection with Pearson correlation. Among the prediction models, the random forest model achieved the highest prediction accuracy. In comparison with the feature selection based on Pearson correlation, MAE and RMSE decreased by 5.0% and 5.1%, respectively, and R^2^ increased by 4.3%. Compared with the full feature-based model, MAE and RMSE are reduced by 1.4% and 1.8%, respectively, and R^2^ is improved by 1.4%.

However, the model proposed in this study also has certain limitations. Although we have improved the prediction accuracy of long-term ozone concentration through feature selection and reduced the dimensionality of the data, the accuracy after dimensionality reduction is still not high. In addition, the stations in the data of this study are mainly distributed in the Northern Hemisphere, and a small number of stations are in the Southern Hemisphere. The monitoring sites include 15 regions, including EUR (Europe), NAM (North America), EAS (East Asia), etc., but they are mainly distributed in NAM (North America), EUR (Europe) and EAS (East Asia). Based on the above limitations, we suggest that (1) more environmental information can be considered as input variables, and the accuracy of predicting long-term ozone concentrations would be further improved by adding more comprehensive information; (2) subsequent research can expand the data coverage by adding more other site information and further study the global long-term ozone concentration characteristics.

## Supplementary Information

Supplementary Tables.

## Data Availability

Data and methods used in the research have been presented in sufficient detail in the study. The dataset is the TOAR database (https://gitlab.jsc.fz-juelich.de/esde/machine-learning/aq-bench/-/blob/master/resources/AQbench_dataset.csv).
