# Supplementary material for: Feature selection for global tropospheric ozone prediction based on the BO-XGBoost-RFE algorithm
Source: Sci Rep. 2022 Jun 2;12:9244. doi: 10.1038/s41598-022-13498-2 (PMC9163069; doi:10.1038/s41598-022-13498-2)
Supplement: Supplementary file 1 — Supplementary Tables. [file 41598_2022_13498_MOESM1_ESM.docx]

# **Supplementary material**

**Supplementary materials to Section 4.1:**

Table 1 Variable names and descriptions

| **Variable** | **Proxy for** |
| --- | --- |
| Country | Emission regulation |
| HTAP region | World region set by the Task Force on Hemispheric  Transport of Air Pollution |
| Climatic zone | Temperature, humidity, radiation |
| Longitude | – |
| Latitude | Radiation, temperature |
| Altitude | Sinks, temperature |
| Relative altitude | Local flow patterns |
| Type | Industry/traffic emissions |
| Type of area | Proximity to human settlement |
| Water in 25 km area | Deposition |
| Evergreen needle leaf forest in 25 km area | VOC emissions, deposition |
| Evergreen broadleaf forest in 25 km area | VOC emissions, deposition |
| Deciduous needle leaf forest in 25 km area | VOC emissions, deposition |
| Deciduous broadleaf forest in 25 km area | VOC emissions, deposition |
| Mixed forest in 25 km area | VOC emissions, deposition |
| Closed shrub lands in 25 km area | VOC emissions, deposition |
| Open shrub lands in 25 km area | VOC emissions, deposition |
| Woody savannas in 25 km area | VOC emissions, deposition |
| Savannas in 25 km area | VOC emissions, deposition |
| Grasslands in 25 km area | VOC emissions, deposition |
| Permanent wetlands in 25 km area | VOC emissions, deposition |
| Croplands in 25 km area | Agricultural emissions |
| Urban and built-up in 25 km area | Human settlement |
| Cropland/natural vegetation mosaic in 25 km area | Emissions, agriculture, deposition |
| Snow and ice in 25 km area | Factor in ozone formation |
| Barren or sparsely vegetated in 25 km area | Emissions, deposition |
| Wheat production | Agricultural emissions |
| Rice production | Agricultural emissions |
| NOx emissions | NOx emissions |
| NO2 full column | NO2 |
| Population density | Human emissions |
| Max population density 5 km | Human emissions nearby |
| Max population density 25 km | Human emissions in area of influence |
| Nightlight 1 km | Industrial activity |
| Nightlight 5 km | Industrial activity nearby |
| Max nightlight 25 km | Industrial activity in area of influence |

**Supplementary materials to Table 1:**

Table 2 All main hyper-parameters of XGBoost

| **Item** | **Hyper-parameter setting** |
| --- | --- |
| Learning_rate | 0.0798 |
| gamma | Minimum split loss is 0.676 |
| Max_depth | Maximum depth of a tree is 8 |
| min_child_weight | Minimum sum of instance weight needed in a child is 4.169 |
| subsample | Subsample ratio of the training instances is default 1 |
| colsample_bytree | the subsample ratio of columns when constructing each tree is 0.7144 |
| reg_lambda | L2 regularization term on weights is 0.2451 |
| reg_alpha | L1 regularization term on weights is 0.4873 |
| sampling_method | Default: uniform |
| colsample_bylevel | the subsample ratio of columns for each level is default 1 |
| colsample_bynode | the subsample ratio of columns for each node (split) is default 1 |
| max_leaves | Maximum number of nodes to be added is default 0 |
| max_delta_step | Maximum delta step we allow each leaf output to be is default 0 |
